# A correlation study of ischiocavernosus muscle injury with different types of pelvic fractures and erectile dysfunction after pelvic fracture

**DOI:** 10.1097/OI9.0000000000000081

**Published:** 2020-09-16

**Authors:** Zongping Chen, Tao Song, Yongxiang Zhuang, Yong Yan, Tong Liu, Kaiyi Mao, Xu Li, Chenghong Zou, Xin Wen, Yuhong Yao, Chao Chen, Sicong Zhao

**Affiliations:** a Department of Urology, Affiliated Hospital of Zunyi Medical University, Zunyi; b Department of Urology, Eastern Hospital of Sichuan Provincial People's Hospital, Chengdu; c Department of Urology, The First Hospital of Putian, Putian; d Department of Urology, Beijing Shijitan Hospital, Capital Medical University, Beijing; e Department of Urology, The Second Affiliated Hospital of Zunyi Medical University, Zunyi, China.

**Keywords:** correlation analysis, erectile dysfunction, ischiocavernosus muscle injury, pelvic fracture

## Abstract

**Objective:**

To explore the correlation between ischiocavernosus muscle injury (ICMI) with different types of pelvic fractures and erectile dysfunction (ED) after pelvic fracture.

**Design:**

Retrospective analysis of a prospective database.

**Setting:**

The study was carried out at the affiliated hospital of Zunyi Medical University.

**Patients/participants:**

A total of 776 male patients with pelvic fracture, aged 18 to 67 years, were recruited for this study by retrospective analysis, and based on the diagnosis of ED and the presence of ICMI, the participants were divided into ED and non-ED groups as well as ICMI and non-ICMI groups.

**Intervention:**

No.

**Main outcome measurements:**

ICMI, the type of pelvic fracture, International Index of Erectile Function-5 scores. Computed tomography/magnetic resonance imaging scans, electromyography (motor unit potential) was used to diagnose ICMI.

**Results:**

The International Index of Erectile Function-5 score was 19.7 ± 5.9. The incidence of ED was 27.3%, the duration time of ED was 30 ± 23 months, and the incidence of reversible ED was 39.6% and of irreversible ED was 60.4%. The incidence of ICMI was 29.4%, among which the incidence of unilateral injury was 57.9%, and the incidence of bilateral injury was 42.1%. Among all pelvic fractures, the incidence of pubic ramus fracture was 88.1%. Bilateral pubic ramus fractures, bilateral fractures of the ischial ramus, and ICMI were independent risk factors for ED after pelvic fracture. Bilateral pubic ramus fractures and pubic symphysis separation were independent risk factors for ICMI. Unilateral ICMI was an independent risk factor for reversible ED, while bilateral ICMI was an independent risk factor for irreversible ED.

**Conclusions:**

ICMI is associated with ED and may be a cause for ED, while pubic ramus fracture, ischial ramus fracture, and pubic symphysis separation may be the main causes of ICMI. Unilateral ICMI may be the main risk factor for transient ED, and bilateral ICMI may be the main risk factor for permanent ED.

## 1 Introduction

ED is the persistent or repeated inability to achieve or maintain sufficient penile erection to sustain a satisfying sexual life.^[1]^ The causes of ED are both organic and psychogenic. ED is a common complication of pelvic fractures, and it has been reported that 3% of cases of ED may result from pelvic fractures or perineal blunt trauma.^[2]^ The incidence of ED ranges from 20% to 84% in patients with urethral injury secondary to perineal trauma or pelvic fractures.[^3^,^4^,^5^,^6^,^7^,^8^,^9^,^10^,^11^] The intimate relationship between the soft tissues and the bony pelvic ring results in a high risk of concomitant local injury associated with fractures of the pelvis.[^12^,^13^,^14^,^15^,^16^] Even without severe urological injury, damage to the delicate vascular and nervous tissues supplying the genitalia can result in sexual dysfunction.[^17^,^18^,^19^,^20^]

The ischiocavernosus muscle (ICM) is a pair of short pinnate muscles attached to the pelvic ring. The ICM arises from the ischial tuberosity and attaches to the pubic ramus, and some muscle fibers enclose the root of the penis.[^21^,^22^,^23^] Contraction of the ICM acts as a lever on the upturned penis, and this lever action of the ICM is suggested to aid in the erectile mechanism by elevating the penile shaft above the horizontal level.^[23]^ However, our study shows that ICM contractions also have the effect of blocking the return of blood to the cavernous body of the penis and maintaining a stiff erection.^[21]^ Moreover, in our series of basic studies on the ICM, it was suggested that cutting off the unilateral ICM in male mice could cause transient erectile dysfunction, resulting in delayed conception in paired female mice, while cutting off the bilateral ICM in male mice could cause permanent erectile dysfunction, resulting in the inability to conceive in paired female mice.^[21]^ After repairing the severed ICM, it was found that some male rats’ erectile function improved, giving their female counterparts the ability to conceive.^[21]^ In our study on the pelvic fracture model caused by external force in male rats, we also confirmed the existence of objective evidence of ischiocavernosus muscle injuries (ICMI) and found that there was relatively little evidence of vascular and nerve injuries, which are related to factors causing erectile dysfunction.^[21]^

We found that ED patients with pelvic fractures had imaging manifestations of ICMI by reviewing their computed tomography (CT)/magnetic resonance imaging (MRI) scans. In this study, we analyzed the correlation and deduced the internal relationship between ICMI and different types of pelvic fractures and ED after pelvic fractures in male patients. This study will provide new ideas for the clinical diagnosis and treatment of ICMI and ED after pelvic fracture.

## 2 Materials and methods

### 2.1 Patients

This study was a retrospective case control study. A consecutive series of data covering 776 male patients with pelvic fractures during the period of January 2010 to December 2018 were collected from the Affiliated Hospital of Zunyi Medical University in China. The inclusion criteria were as follows: male; traumatic pelvic fracture; age range of 18 to 70 years; normal secondary sexual characteristics; normal erectile function before pelvic fracture; complete and accurate clinical records (hospitalizations from January 1, 2010, to December 31, 2018); and follow-up visits lasting for 1 to 10 years. The exclusion criteria were as follows: pelvic fracture with brain trauma; pelvic fracture with spinal trauma; death from a fractured pelvis; pelvic fractures associated with hypertension, diabetes, prostatic hyperplasia, and delayed gonadal dysfunction; pathological pelvic fracture; incomplete clinical records; and patients with pelvic fracture who refused follow-up visits. The institutional review board of the Affiliated Hospital of Zunyi Medical University approved the present study in September 2018, and all procedures performed in the present study involving human participants were in accordance with the ethical standards of the institutional committee and with the 1964 Helsinki Declaration and its later amendments or comparable ethical standards.

### 2.2 Blood tests

To exclude hormonal factors related to ED, the following testosterone levels were measured in peripheral blood plasma by radioimmunoassay: serum total testosterone (TT) and free testosterone (FT), estradiol (E2), luteinizing hormone (LH), and follicle-stimulating hormone (FSH). To exclude metabolic factors related to ED, fasting plasma glucose (FPG), triglyceride (TG), high-density lipoprotein cholesterol (HDL-C), low-density lipoprotein cholesterol (LDL-C), and total cholesterol (TC) levels were determined with a fully automatic biochemical analyzer.

### 2.3 International index of erectile function-5 (IIEF-5) questionnaires

IIEF-5 questionnaires were completed by all patients. The method was used to categorize ED as follows: no ED (scores of 22–25) or ED (scores of 5–21) according to the literature.^[24]^

### 2.4 Diagnosis of pelvic fracture

Pelvic fracture was diagnosed by X-ray and CT.

### 2.5 Diagnosis and evaluation criteria of ICMI

CT/MRI/MUP was used to diagnose ICMI. The diagnostic criteria of ICMI include at least 2 of the following 4 indicators: Avulsion of the starting tendon of the ICM; ICM swelling, hematoma formation, starting tendon avulsion injury, and the fracture end embedded into the ICM; In the chronic phase, the impaired ICM demonstrated a speckle formation on T2WI showing low signal, and muscle atrophy and fat infiltration on T1WI showing high signal; ICM showed a shortened mean time limit, decreased amplitude and increased multiphase wave at the motor unit potential, or showed a shortened average time limit, decreased amplitude and increased multiphase waves at the motor unit potential.

### 2.6 Evaluation criteria of reversible/irreversible ED

Evaluation criteria of reversible ED: the course of ED recovered spontaneously within 12 months or with conventional medication within 12 months.

Evaluation criteria of irreversible ED: ED lasted longer than 12 months or did not recover after conventional medication.

### 2.7 Data extraction

The following parameters were obtained from each participant: age (year); blood pressure; TT, FT, E2, FSH, LH, FPG, TG, TC, and HDL-C levels; IIEF-5 score; presence of ED; type of ED, including reversible and irreversible ED; duration time of ED; presence of ICMI; type of ICMI, including unilateral injury and bilateral injury; type of pelvic fracture and treatment of pelvic fracture; and presence of posterior urethral injury, bladder injury and rectal injury.

### 2.8 Statistical analyses

Statistical analyses were performed using Statistical Package for the Social Sciences (SPSS Inc, Chicago, Illinois) version 18.0 for windows. The selected characteristics are expressed as the mean and standard deviation (mean ± SD) as well as the percentage (%). Multivariate logistic regression analysis and chi-square test were used, and the multivariate-adjusted odds ratios (ORs) and 95% confidence intervals (CIs) were simultaneously estimated. Differences were considered statistically significant with a *P* value of < .05.

## 3 Results

### 3.1 Clinical baseline characteristics of male patients with pelvic fractures

All of the patients had normal levels of blood hormones, including TT, FT, E2, LH, and FSH. Blood lipids, including TG, TC, LDL-C and HDL-C, blood glucose (FPG), and blood pressure levels, were also normal in these patients (data not shown). The principal characteristics of our study population of male patients with pelvic fractures are listed in Table 1. A total of 776 men were analyzed, and the mean follow-up period was 67 (range, 12–120) months. The average age was 46 ± 13 (range, 18–67) years. The IIEF-5 score was 19.7 ± 5.9 (range, 5–25). The incidence of ED was 27.3% (212/776), the duration time of ED was 30 ± 23 months, and the incidence of reversible ED was 39.6% (84/212) and of irreversible ED was 60.4% (128/212). The incidence of ICMI was 29.4% (228/776), among which the incidence of unilateral injury was 57.9% (132/228) and of bilateral injury was 42.1% (96/228). Among all pelvic fractures, the incidence of pubic ramus fracture was 88.1% (684/776), pubic body fracture was 12.1% (94/776), ilium fracture was 37.4% (290/776), coccyx fracture was 0.3% (2/776), fracture of the ischium was 8.5% (66/776), fracture of ischial ramus was 10.7% (83/776), pubic symphysis separation was 9.2% (71/776), sacrum fracture was 36.5% (283/776), simple fracture was 1.8% (14/776), and complex fracture was 98.2% (762/776). Furthermore, 73.7% (572/776) of male patients with pelvic fractures were treated conservatively, while 26.3% (204/776) were treated surgically. Posterior urethral injury accounted for 7.1% (55/776), bladder injury accounted for 2.4% (19/776), and rectal injury accounted for 0.8% (6/776).

**Table 1:**
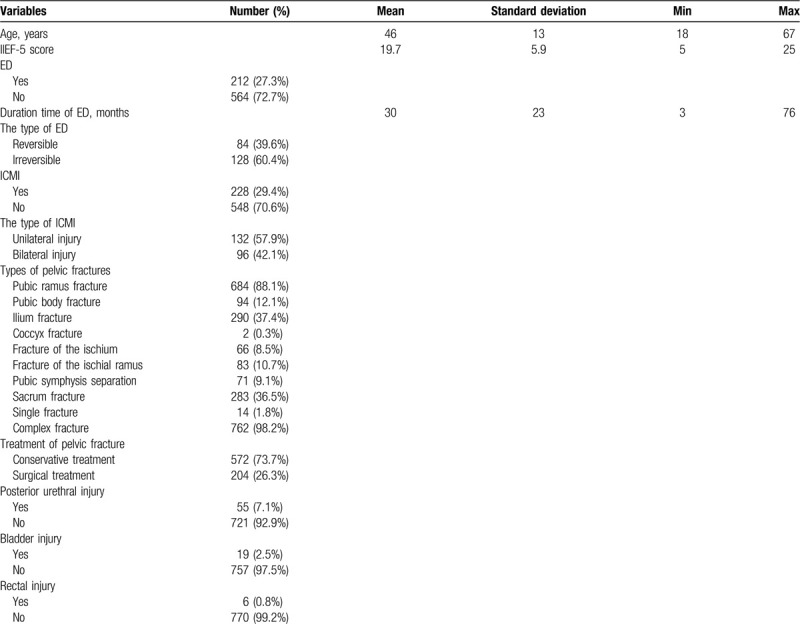
The baseline characteristics of 776 male patients with pelvic fractures.

### 3.2 The analysis of the related factors of erectile dysfunction after pelvic fracture

In this study, we conducted a correlation analysis of the effects of different types of pelvic fractures, treatment options of pelvic fractures, ICMI, posterior urethral injury, bladder injury, and rectal injury on erectile dysfunction. As shown in Table 2, we found that bilateral pubic ramus fractures (OR = 15.1, 95% CI: 6.4–35.6, *P* < .001), bilateral fractures of the ischium (OR = 1.2, 95% CI: 1.1–1.6, *P* = .006), bilateral fractures of the ischial ramus (OR = 17.9, 95% CI: 2.7–19.2, *P* = .003), pubic symphysis separation (OR = 2.7, 95% CI: 1.0–7.2, *P* = .038), unilateral ICMI (OR = 16.8, 95% CI: 2.9–48.4, *P* < .001), bilateral ICMI (OR = 26.0, 95% CI: 5.5–122.9, *P* < .001), and posterior urethral injury (OR = 3.7, 95% CI: 1.3–10.5, *P* = .015) were correlated with the occurrence of ED after pelvic fractures and were independent risk factors for ED after pelvic fractures. However, the presence of ED after pelvic fracture was not significantly correlated with unilateral pubic ramus fracture (OR = 0.4, 95% CI: 0.2–1.0, *P* = .058), unilateral pubic body fracture (OR = 0.5, 95% CI: 0.3–1.0, *P* = .067), bilateral pubic body fracture (OR = 0.4, 95% CI: 0.1–1.2, *P* = .095), unilateral ilium fracture (OR = 1.6, 95% CI: 1.1–2.2, *P* = .112), bilateral ilium fracture (OR = 1.9, 95% CI: 0.7–5.0, *P* = .189), coccyx fracture (OR = 2.4, 95% CI: 0.1–38.7, *P* = .545), unilateral fracture of the ischium (OR = 0.4, 95% CI: 0.1–3.3, *P* = .429), unilateral fracture of the ischial ramus (OR = 0.6, 95% CI: 0.3–1.5, *P* = .290), sacrum fracture (OR = 1.0, 95% CI: 0.5–1.7, *P* = .884), surgical treatment of pelvic fractures (OR = 1.7, 95% CI: 0.9–3.4, *P* = .127), bladder injury or rectal injury.

**Table 2:**
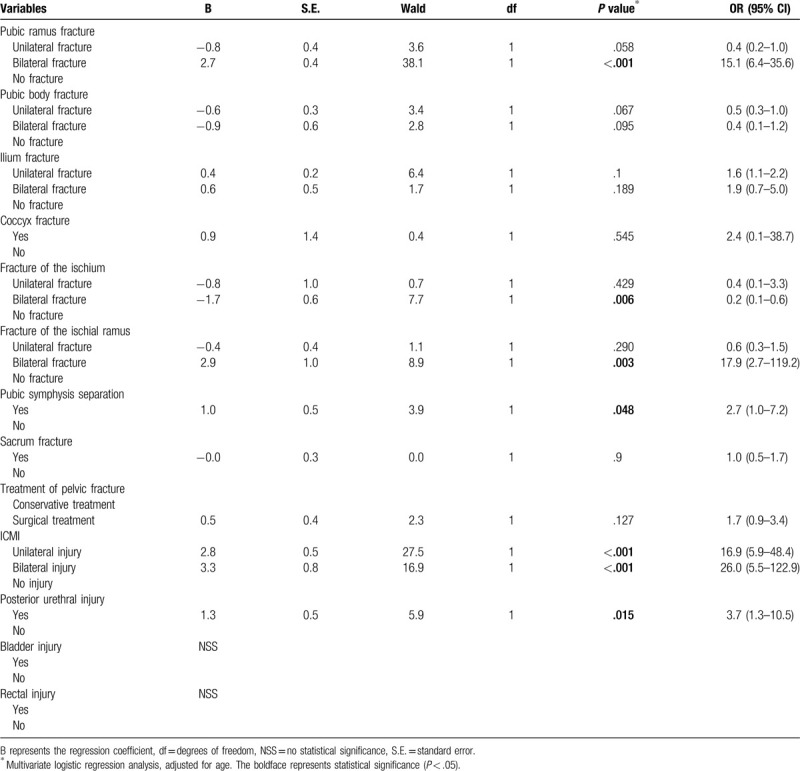
The analysis of the related factors of erectile dysfunction after pelvic fracture.

### 3.3 The analysis of the related factors of ischiocavernosus muscle injury after pelvic fracture

In this study, we conducted correlation analyses of the effects of different types of pelvic fractures, treatment options for pelvic fractures, ED, posterior urethral injury, bladder injury, and rectal injury on ICMI. As shown in Table 3, we found that bilateral pubic ramus fractures (OR = 43.8, 95% CI: 9.3–206.5, *P* < .001), bilateral pubic body fractures (OR = 20.6, 95% CI: 4.6–92.5, *P* < .001), pubic symphysis separation (OR = 10.4, 95% CI: 4.4–24.8, *P* < .001), the surgical treatment of pelvic fracture (OR = 4.1, 95% CI: 2.5–6.7, *P* < .001), the occurrence of ED (OR = 14.8, 95% CI: 5.9–43.3, *P* < .001), the occurrence of reversible ED (OR = 15.9, 95% CI: 5.7–43.4, *P* < .001), and the occurrence of irreversible ED (OR = 25.5, 95% CI: 5.5–112.4, *P* < .001) were correlated with ICMI and were independent risk factors for ICMI. However, ICMI was not significantly correlated with unilateral pubic ramus fracture (OR = 4.1, 95% CI: 0.9–19.3, *P* = .079), unilateral pubic body fracture (OR = 0.7, 95% CI: 0.1–5.5, *P* = .706), unilateral ilium fracture (OR = 1.4, 95% CI: 1.1–2.2, *P* = .122), bilateral ilium fracture (OR = 0.4, 95% CI: 0.1–1.2, *P* = .085), coccyx fracture (OR = 2.4, 95% CI: 0.1–37.8, *P* = .545), unilateral fracture of the ischium (OR = 0.5, 95% CI: 0.2–1.4, *P* = .171), bilateral fractures of the ischium (OR = 0.5, 95% CI: 0.1–4.0, *P* = .488), unilateral fracture of the ischial ramus (OR = 1.5, 95% CI: 0.8–3.0, *P* = .229), bilateral fractures of the ischial ramus (OR = 0.3, 95% CI: 0.0–1.8, *P* = .182), sacrum fracture (OR = 1.0, 95% CI: 0.6–1.6, *P* = .963), posterior urethral injury (OR = 0.9, 95% CI: 0.3–2.3, *P* = .790), bladder injury or rectal injury.

**Table 3:**
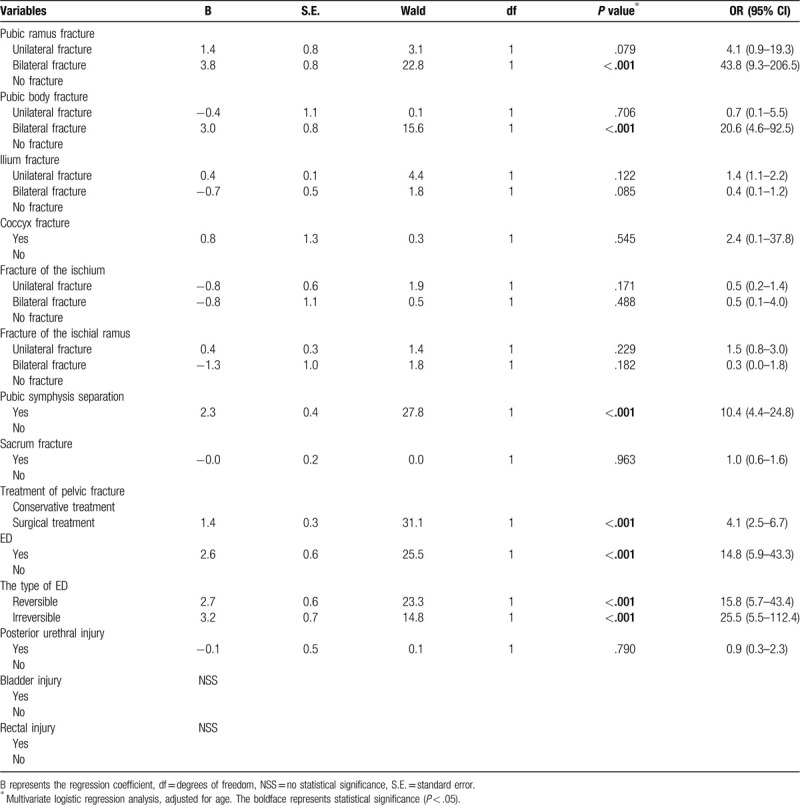
The analysis of the related factors of ischiocavernosus muscle injury after pelvic fracture.

### 3.4 The correlation analysis of different types of ischiocavernosus muscle injuries and different types of erectile dysfunction after pelvic fracture

In this study, we conducted a correlation analysis of different types of ischiocavernosus muscle injuries and different types of erectile dysfunction after pelvic fracture. As shown in Table 4, we found that unilateral ICMI was correlated with reversible ED, and unilateral ICMI (OR = 2.9, 95% CI: 1.3–6.3, *P* = .009) was an independent risk factor for reversible ED, while bilateral ICMI was correlated with irreversible ED, and bilateral ICMI (OR = 25.4, 95% CI: 7.2–89.6, *P* < .001) was an independent risk factor for irreversible ED.

**Table 4:**
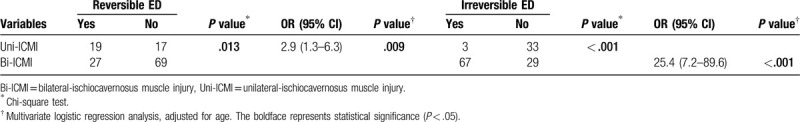
The correlation analysis of different types of ischiocavernosus muscle injuries and different types of erectile dysfunction after pelvic fracture.

## 4 Discussion

In this study, we evaluated the correlation among ICMI, pelvic fracture types, and ED after pelvic fracture. The results showed that the incidence of ED was 27.3%, the duration time of ED was 30 ± 23 months, and the incidence of reversible ED was 39.6% and of irreversible ED was 60.4%. The incidence of ICMI was 29.4%, among which the incidence of unilateral injury was 57.9%, and the incidence of bilateral injury was 42.1%. Among all pelvic fractures, the incidence of pubic ramus fracture was 88.1%, and the incidence of complex fracture was 98.2%. In addition, we found that bilateral pubic ramus fractures, bilateral fractures of the ischial ramus, pubic symphysis separation, unilateral ICMI, bilateral, and posterior urethral injury were correlated with the occurrence of ED after pelvic fractures and were independent risk factors for ED after pelvic fractures. At the same time, we also found that bilateral pubic ramus fractures, bilateral pubic body fractures, pubic symphysis separation, the surgical treatment of pelvic fracture, and the occurrence of ED/reversible ED/irreversible ED were correlated with ICMI and were independent risk factors for ICMI. Through further analysis, we found that unilateral ICMI was correlated with reversible ED, and unilateral ICMI was an independent risk factor for reversible ED, while bilateral ICMI was correlated with irreversible ED, and bilateral ICMI was an independent risk factor for irreversible ED. Therefore, we believe that ICMI is associated with ED and may be a cause for ED, while pubic ramus fracture and pubic symphysis separation may be the main causes of ICMI.

**Figure 1:**
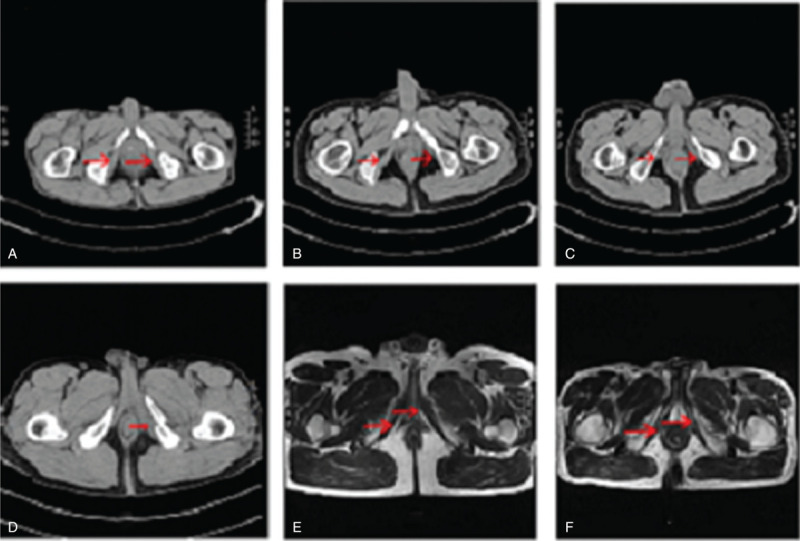
The appearance of ischiocavernosus muscle injury is shown by computed tomography/magnetic resonance imaging after pelvic fracture. (A) The normal ICM; (B, C) avulsion of the starting tendon of the ICM; (D) ICM swelling, hematoma formation, starting tendon avulsion injury, and the fracture end embedded into the ICM; (E, F) in the chronic phase, the impaired ICM demonstrated a speckle formation on T2WI showing low signal, and muscle atrophy and fat infiltration on T1WI showing high signal (→ was shown in). The area indicated by the red arrow is ICM injuries.

**Figure 2:**
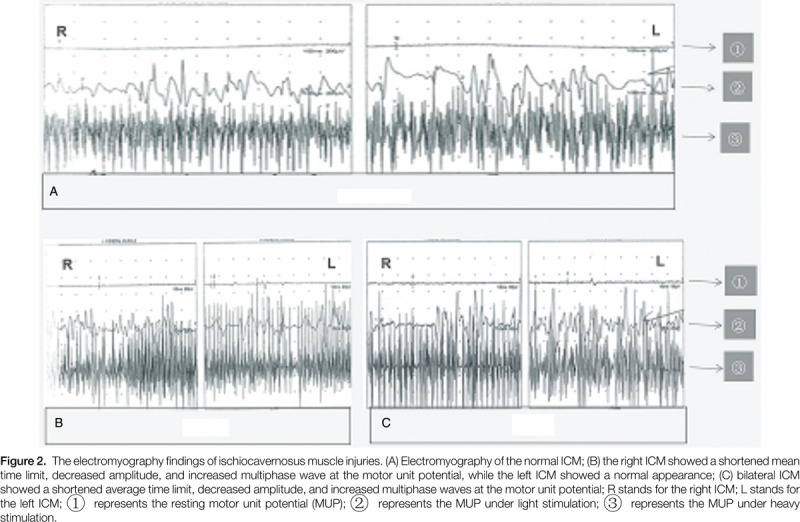
The electromyography findings of ischiocavernosus muscle injuries. (A) Electromyography of the normal ICM; (B) the right ICM showed a shortened mean time limit, decreased amplitude, and increased multiphase wave at the motor unit potential, while the left ICM showed a normal appearance; (C) bilateral ICM showed a shortened average time limit, decreased amplitude, and increased multiphase waves at the motor unit potential; R stands for the right ICM; L stands for the left ICM; 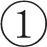 represents the resting motor unit potential (MUP); 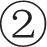 represents the MUP under light stimulation; 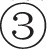 represents the MUP under heavy stimulation.

The above results obtained by us are quite different from previous research results of other scholars. Previous studies have shown that ED after pelvic fractures is mainly caused by nerve injury and blood vessel injury. Of course, there are also psychogenic factors.[^5^,^25^,^26^,^27^] Similarly, in terms of the role of the ICM in the mechanism of penile erection, other scholars believe that when the penis is erect, the contraction of this muscle mainly plays a role in the upturned penis.[^23^,^28^,^29^] Through a series of basic studies, we found that the ICM plays an important role in the mechanism of penile erection, that is, the contraction of this muscle can block the blood vessels of the cavernous body located at the root of the penis, prevent the return of blood to the cavernous body, and maintain the hard erection of the penis, thus playing an important role.^[21]^ In our study of the pelvic fracture model caused by external force in male rats, we also confirmed the existence of objective evidence for ICM injury and found that there was relatively little evidence of vascular and nerve injuries, which are related to factors causing erectile dysfunction.^[21]^ In clinical work, we focused on the manifestations of ICMI in the CT/MRI examination of the pelvis in patients with ED after pelvic fracture (see Fig. 1) and performed ICM electromyography examination on some patients, which resulted in abnormal electromyography evidence (see Fig. 2). Meanwhile, in this study, nerve evoked potential detection was performed on the adjacent muscles of ICM, including the bulbar spongiform muscle and the muscles of the medial thigh, and the results indicated no significant nerve damage (data not shown). This is the main reason why we conducted this study, and indeed, through this study, we proved the possibility of our view on the relationship between ICMI and different types of pelvic fractures and ED after pelvic fractures. Our findings are expected to attract the attention of colleagues, and in clinical work, we expect to focus on the diagnosis of ICMI-induced ED in male patients with pelvic fractures and conduct further research with follow-up treatment.

The present study has some limitations. In this study, we did not perform a nocturnal penile tumescence test, duplex ultrasonography of the penis, or cavernography of the penis. These examinations are currently recommended etiological examination methods for determining ED, and we only analyzed the correlation between all types of ED after pelvic fracture and ICMI. This results in a certain deficiency that needs to be further explained. However, this also gives us a direction for our future research.

In conclusion, in this study, we systematically analyzed the correlation among ICMI, types of pelvic fractures and ED after pelvic fractures through large clinical sample data. Our results showed that pubic ramus fracture, ischial ramus fracture, and pubic symphysis separation were strongly correlated with ICMI, and ICMI was strongly correlated with ED after pelvic fracture. Moreover, pubic ramus fracture, ischial ramus fracture, and pubic symphysis separation were independent risk factors for ICMI, and ICMI was an independent risk factor for ED after pelvic fracture. Therefore, we conclude that ICMI is associated with ED and may be a cause for ED, while pubic ramus fracture, ischial ramus fracture, and pubic symphysis separation may be the main causes of the high credibility and high clinical value of ICMI.
